# Familial Exudative Vitreoretinopathy

**DOI:** 10.4274/tjo.67699

**Published:** 2015-08-05

**Authors:** Selçuk Sızmaz, Yoshihiro Yonekawa, Michael T. Trese

**Affiliations:** 1 Çukurova University Faculty of Medicine, Department of Ophthalmology, Adana, Turkey; 2 Associated Retinal Consultants Pc, Royal Oak, Michigan, USA

**Keywords:** Familial exudative vitreoretinopathy, NDP, FZD, LRP5, TSPAN12

## Abstract

Familial exudative vitreoretinopathy (FEVR) is a hereditary disease associated with visual loss, particularly in the pediatric group. Mutations in the NDP, FZD4, LRP5, and TSPAN12 genes have been shown to contribute to FEVR. FEVR has been reported to have X-linked recessive, autosomal dominant, and autosomal recessive inheritances. However, both the genotypic and phenotypic features are variable. Novel mutations contributing to the disease have been reported. The earliest and the most prominent finding of the disease is avascularity in the peripheral retina. As the disease progresses, retinal neovascularization, subretinal exudation, partial and total retinal detachment may occur, which may be associated with certain mutations. With early diagnosis and prompt management visual loss can be prevented with laser photocoagulation and anti-VEGF injections. In case of retinal detachment, pars plana vitrectomy alone or combined with scleral buckling should be considered. Identifying asymptomatic family members with various degrees of insidious findings is of certain importance. Wide-field imaging with fluorescein angiography is crucial in the management of this disease. The differential diagnosis includes other pediatric vitreoretinopathies such as Norrie disease, retinopathy of prematurity, and Coats’ disease.

## INTRODUCTION

Familial exudative vitreoretinopathy (FEVR) was first described by Criswick and Schepens^1^ in 1969. The characteristic clinical features were based on peripheral retinal vascular changes of heterogenic severity. Those changes included avascular peripheral retina, peripheral retinal neovascularization, macular dragging, subretinal exudation, vitreoretinal traction, and retinal detachment. The vascular features were first demonstrated by fluorescein angiography (FA) by Canny and Oliver^2^ in 1976. In 1998, Pendergast and Trese^3^ described a classification scheme, which was recently updated,^4^ for FEVR (Table 1). Clinical presentation may vary between asymptomatic avascular peripheral retina and total retinal detachment that leads to vision loss. Although the disease is known to be familial, the phenotypic features can still be variable within families.^5,6,7^

Familial exudative vitreoretinopathy is a lifelong disease that can progress, resulting in blindness despite treatment. The presentation and course of the disease show variability, making the diagnosis and management challenging.^5^

## INHERITANCE AND PATHOPHYSIOLOGY

A positive family history is reported to be present in approximately 20-40% of cases.8 The disease is currently known to be inherited in three forms: autosomal dominant (AD), autosomal recessive (AR), and X-linked recessive (XR), with the AD form being the most common. However, the inheritance patterns and expressivity show a heterogeneous course, as do the clinical features and prognosis of the disease.^6,9^

Mutations in four genes related to FEVR have been described. These gene products are Norrin Disease Protein (NDP), Frizzled-4 (FZD4), low-density lipoprotein receptor-related protein 5 (LRP5), and tetraspanin-12 (TSPAN12). The NDP gene is located on the X chromosome (Xp11.4), FZD4 and LRP5 are located on chromosome 11 (11q14.2 and 11q13.2, respectively), and TSPAN12 is located on chromosome 7 (7q31.31). Mutations in the causative genes and the resultant diseases are as follows: NDP gene, X-linked recessive FEVR or Norrie disease; FZD4 gene, AD FEVR; LRP5 gene, both AD and AR FEVR; and TSPAN12, AD FEVR.10,11,12,13,14 The protein products of these genes are involved in the canonical Wnt and Norrin signaling pathways. The Wnt signaling pathway is essential for the organogenesis and angiogenesis of the mammalian eye. Norrin, which is coded by NDP, exerts a strong affinity to receptor FZD4. In this pathway, LRP5 is the co-receptor of FZD4. The complex of the ligand and the receptors is mediated by TSPAN12, an auxiliary transmembrane protein, which binds to Norrin multimers resulting in enhancement of FZD4 multimerization and clustering. Norrin-receptor complex activates the canonical Wnt pathway.^9,10,11,15,16^ In case of a blockade in the Norrin binding pathway, the signaling is disturbed, resulting in a depression in target protein production.^15^ Although the NDP mutations are involved both in Norrie disease and FEVR, most mutations are related to Norrie disease, whereas a smaller percentage of these mutations cause X-linked FEVR. Mutations in NDP were also reported to be associated with persistent fetal vasculature syndrome, Coats’ disease, and retinopathy of prematurity (ROP).^11,12^

Mutations in the FEVR-related genes were found in 60% of Dutch families with FEVR. The rate of FZD4 mutations was 25%.^13^ Twelve FZD4 mutations were found to be involved in 31.3% of Chinese patients with FEVR. In this study, the course of FEVR was more severe in patients who were found to have more than one mutation of the FZD4 gene, compared with those who had one mutation. This finding led the authors to speculate that more complex genotypes are associated with more severe phenotypes.^9^ In a report from Japan, the rate of NDP mutations in patients with FEVR was 6%.^17^ In another study, the mutation rate of FZD4 was 18%, half of which were novel mutations. The authors reported significant intrafamily variability, with severe and subtle forms between the members of the same family. The authors also concluded that the type of the mutation (missense, deletion, insertion, or stop) was not correlated with the disease severity.^10^ Mutations of FZD4 were implicated in 5-40% of FEVR families, while this rate was 12-18% for LRP5 gene mutations.^18^ Mutations in TSPAN12 were encountered in 10% of FEVR cases. The authors found that disease severity worsened as the number of mutant alleles increased; patients with two affected alleles exhibited severe findings.^15^ Similarly, two patients both carrying two LRP5 mutations demonstrated more severe clinical features when compared with their parents.^19^ A double sequence change in the FZD4 gene was also reported.^20^ However, the ocular manifestations of mutations in all four genes do not show significant differences.^21^

FEVR patients with mutations in both FZD4 and LRP5 have been reported; however, this association was not reported to cause increased severity in the phenotype.^11,15^ Shastry and Trese22 reported that a Factor V-Leiden mutation cosegregated with the FZD4 mutation in a FEVR family.

Boonstra et al.^13^ estimated the penetrance of FEVR to be 90%. Non-penetrance could also be present in approximately one-quarter to one-third of mutation carriers.^11,23,24^

Given the variability in the heredity of the disease, one could argue that the large spectrum of the clinical features would not be surprising.

## CLINICAL FEATURES AND DIAGNOSIS

The diagnosis of FEVR should be based on the following entities:

- Lack of peripheral retinal vascular development in at least one eye,

- Lack of history of prematurity or in a preterm born, a disease tempo not consistent with ROP,

- Variable degrees of vitreoretinal traction, subretinal exudation, or retinal neovascularization occurring at any age.^8^

The most prominent and common finding of FEVR is the avascular peripheral retina, which is a result of premature retinal angiogenesis due to the aforementioned mutations in the genes related to the organogenesis of the eye.^13,25^ Of course, this should be considered to be the most prominent and common finding, as it is hard to detect all patients at this stage. Macular dragging, radial retinal folds, retinal neovascularization, preretinal vitreous organization, vitreoretinal proliferation, subretinal exudation, and retinal detachment can be present in various combinations. Vitreoretinal adhesions, venous-venous anastomoses, supernumerous vascular branching, and V-shaped retino-choroidal degeneration could be presented in milder forms of the disease, as well as peripheral avascularity. Retinal vessels, both arteries and veins, demonstrate excessive branching with the bifurcations having right angles.

In advanced stages of the disease, as the manifestations become more severe, neovascularization, sub/intraretinal hemorrhages and exudates, vascularized preretinal membranes that can lead to retinal folds, macular dragging, and retinal detachment occur. Capillary non-perfusion due to avascularity and pathologic vitreoretinal adhesions make the scaffold for these advanced features. 

The disease does not necessarily follow the stages sequentially, and the eyes do not always present with symmetrical findings. Some features strongly indicate FEVR, such as a knife-like radial retinal fold extending from the optic nerve to even the ciliary body. However, a careful examination is mandatory.^6,8,26^ Retinoschisis is not an irrelevant finding either.^26^ Besides tractional retinal detachment (RD), rhegmatogenous RD could also complicate the clinical presentation.^27^

A positive family history will assist making the diagnosis. However, a negative family history would not exclude FEVR, as novel mutations can also occur. On the other hand, because of the phenotypic heterogeneity, it is important to identify asymptomatic family members. Kashani et al.^6^ reported that in asymptomatic family members of FEVR patients, 58% had stage 1 and 35% had stage 2 FEVR.

**Figure 1:**
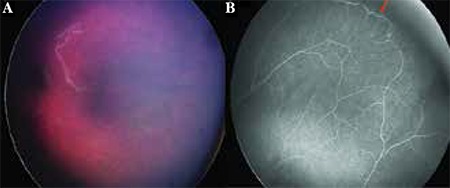
Stage 1 familial exudative vitreoretinopathy (FEVR): (A) RetCam (Clarity Medical Systems, Pleasanton, California, USA) color fundus photography shows that the appearance of stage 1 disease may appear deceivingly normal on funduscopy. (B) However, wide-field fluorescein angiography demonstrates abnormal peripheral vasculature and nonperfusion. Venous-venous vascular loops (arrow) are characteristic of FEVR

**Figure 2:**
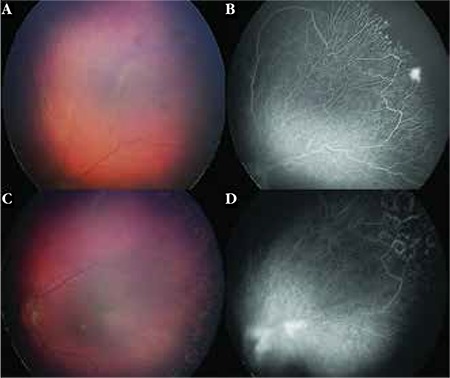
Stage 2B familial exudative vitreoretinopathy (FEVR): (A) RetCam color fundus photography shows mild venous dilation and tortuosity and vascular loops that are much more evident with angiography. (B) Wide-field fluorescein angiography clearly demonstrates the abnormal vascular patterns including venous-venous looping, branching vessels with bulb-like telangiectatic endings, focus of leakage, and peripheral nonperfusion. (C-D) Laser treatment was provided to the avascular peripheral retina, which resulted in resolution of vascular leakage

**Figure 3:**
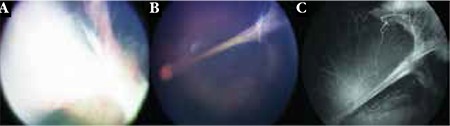
This patient presented with Stage 5 familial exudative vitreoretinopathy (FEVR) in the right eye, characterized by complete retinal detachment (A), and Stage 4B disease in the left eye (B). The left eye has a knife-like retinal fold with peripheral avascularity with abnormal vessels and leakage (C). Notice the retinal pigment epithelial alterations at the base of the fold

Currently, for accurate diagnosis, a thorough examination combined with wide field fluorescein angiography is essential (Figures 1, 2, 3). Fluorescein angiography enhances the diagnostic sensitivity because subtle vascular changes in the periphery or even in the posterior pole can easily be overlooked by conventional funduscopy. Wide-field retinal imaging would also be helpful to detect asymptomatic family members of index patients. The incidence of the disease could be higher than expected. The determination of asymptomatic family members with the disease at child-bearing age is essential for genetic counseling and neonatal screening.^6^ The disease tends to become quiescent in the late teens or early twenties; however, later recurrences frequently occur with an unpredictable clinical course.^6^

## DIFFERENTIAL DIAGNOSIS

Distinguishing FEVR from Norrie disease, ROP and Coats’ disease may be challenging.^8^ As NDP gene mutations are implied in the pathogenesis of both diseases, FEVR must be differentiated from Norrie disease. Deletion and truncation mutations in the gene result in Norrie disease without exception. However, missense mutations cause either Norrie disease or FEVR. In Norrie disease, retinal vascular anomalies could be accompanied by mental retardation and hearing loss in approximately in a quarter of patients. Besides genetic alterations, FEVR is differentiated from Norrie disease by its slow progression.^8,17^ Mutations involving cysteine residues of the NDP gene resulted in Norrie disease, whereas mutations involving the non-cysteine residues were found to be associated with FEVR.^28^ As well as Norrie disease, persistent fetal vasculature syndrome, which has quite similar phenotypic features with Norrie disease without evidence of inheritance, must be considered in differential diagnosis. However, unlike FEVR, both entities lack preretinal neovascularization despite a severely avascular peripheral retina.^29^

Unlike ROP, FEVR patients are full-term infants. ROP frequently shows spontaneous regression; although there are sequelae, late recurrences do not occur.^8^ However, there is conflicting data about the role of the NDP gene in ROP.^30^ Recently, the same FZD4 mutations were reported in both diseases, making the differential diagnosis complex. FZD4 mutations were reported to contribute to aggressive posterior ROP.^16^

When first reported by Criswick and Schepens,^1^ the presenting features of FEVR were briefly described; however, although the authors suggested the inherited characteristics of the disease, they could not define an inheritance pattern. As the patients to whom they referred did not have history of prematurity, the authors mainly considered Coats’ disease in the differential diagnosis and distinguished the entity from Coats’ disease by the family history, peripheral nature of the disease, and the more severe involvement of the vitreous. Unlike Coats’, male preponderance is not characteristic for FEVR.^31^ Recently, Robitaille et al.^10^ reported the disparity between Coats’ disease and FZD4 mutations.

## TREATMENT

Stage 1 FEVR requires no treatment but regular follow-up. The primary treatment of FEVR is laser photocoagulation. The peripheral avascular areas should be ablated with laser in case of neovascularization demonstrating leakage in FA, which can even be performed in fractionated sessions. Successful outcome with laser treatment was reported even in stage 4 FEVR patients. Cryotherapy was used for treatment of neovascularization, particularly in patients with small, non-dilating pupils or with hazy media; however, this is no longer considered an option. In patients with RD, surgical intervention should be considered. In this case, scleral buckle alone can help in cases with limited detachment; however, more severe cases would necessitate pars plana vitrectomy (PPV). Earlier diagnosis and management would result in better prognosis.^3,25^

In a single study, based on the finding that dysregulation of the canonical Wnt pathway was associated with increased vascular endothelial growth factor (VEGF) levels, intravitreal injection of an anti-VEGF agent (pegaptanib sodium) was administered in persistent FEVR cases and reduced exudation and vascular activity were achieved.^5^ Recently, intravitreal bevacizumab was also reported to cause an immediate regression of neovascularization.^32^

In cases that undergo PPV for tractional RD, the taut nature of the posterior hyaloid must be considered. Intravitreal injection of autologous plasmin was suggested for better cleavage of the vitreomacular interface and enhanced surgical dissection of the vitreous sheets from the retinal surface in these patients.^33^ Although scleral buckling would help reduce traction, the main advantage of vitrectomy is removing the proliferation consisting of membranes and proliferative tissue and laser photocoagulation of the avascular area can be beneficial.^27^

In their series, Kulaçoğlu et al.^34^ reported 5 cases of FEVR. All patients were presented at various stages of the disease, demonstrating asymmetric presentation. However, all 5 patients lacked a history of premature birth. Only one patient had a positive family history and all cases were late referrals with poor prognosis. In our opinion, their case series from the Turkish literature is in accordance with the reported features of the disease.

## Figures and Tables

**Table 1:**
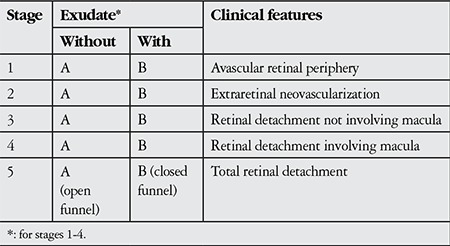
Updated clinical classification of familial exudative vitreoretinopathy^4^
